# Tasks and responsibilities in physical activity promotion of older patients during hospitalization: A nurse perspective

**DOI:** 10.1002/nop2.588

**Published:** 2020-08-30

**Authors:** Kira Scheerman, Joram Willem Mesters, Jay Noël Borger, Carel Gerardus Maria Meskers, Andrea Britta Maier

**Affiliations:** ^1^ Section of Gerontology and Geriatrics Department of Internal Medicine Amsterdam UMC Vrije Universiteit Amsterdam Amsterdam The Netherlands; ^2^ Amsterdam Movement Sciences Amsterdam The Netherlands; ^3^ Rehabilitation Medicine Amsterdam UMC Vrije Universiteit Amsterdam Amsterdam The Netherlands; ^4^ Department of Human Movement Sciences, @AgeAmsterdam Vrije Universiteit Amsterdam Amsterdam The Netherlands; ^5^ Department of Medicine and Aged Care Royal Melbourne Hospital @AgeMelbourne University of Melbourne Melbourne Vic. Australia

**Keywords:** aged, hospitalization, mixed method, nurses, physical activity

## Abstract

**Aim:**

To investigate how nurses perceive tasks and responsibilities in physical activity promotion of hospitalized older patients and which factors are of influence.

**Design:**

Mixed methods sequential explanatory design.

**Methods:**

One hundred and eight nurses participated in a questionnaire survey and 51 nurses in a subsequent in‐depth interview. Data were collected on tasks and responsibilities in physical activity promotion and their influencing factors as perceived by nurses. Quantitative data were analysed using descriptive statistics and a deductive approach with directed content analysis was used for the data from the interviews.

**Results:**

Nurses perceived to have a dominant role in physical activity promotion of older patients during hospitalization. Ninety per cent of the nurses stated to be responsible for physical activity promotion and 32% stated to be satisfied with the actual level of physical activity of their patients. Nurses have specified to be responsible for signalling and performing physical activity promotion tasks and had final responsibility for transfers from bed to chair and promotion of daily activities. Influencing factors were low patient motivation, high workload causing priority shifts of tasks and the role of physicians.

## 1 INTRODUCTION

More than one third of all hospital discharges in the United States affects patients of 65 years or older (Data from Healthcare Cost and Utilization Project database 2013). Older hospitalized patients spend most of the time lying in bed (Brown, Redden, Flood, & Allman, 2009; Callen, Mahoney, Grieves, Wells, & Enloe, 2004; Fisher et al., 2011; Pedersen et al., 2013; Sallis et al., 2015) while physical inactivity in this group is associated with functional decline (Brown, Friedkin, & Inouye, 2004; Zisberg et al., 2011), readmissions (Fisher, Graham, Ottenbacher, Deer, & Ostir, 2016), nursing home admissions and death (Brown et al., 2004).

The level of physical activity during hospitalization has been indicated as a modifiable risk factor for complications related to hospitalization (Zisberg, Shadmi, Gur‐Yaish, Tonkikh, & Sinoff, 2015). Physical activity, including bed mobility, transfers, activities of daily living and walking, was found to have positive effects on physical and psychosocial outcomes during and after hospitalization (Resnick & Boltz, 2019). Increasing physical activity levels during hospitalization was found to reduce length of stay (McCullagh, Dillon, Dahly, Horgan, & Timmons, 2016), and a threshold of 900 steps a day during hospitalization was indicated to prevent functional decline (Agmon et al., 2017). Patient‐related organizational and environmental factors have been shown to contribute to physical activity promotion of hospitalized older patients (Boltz, Capezuti, & Shabbat, 2011; Brown, Williams, Woodby, Davis, & Allman, 2007; Doherty‐King & Bowers, 2011; Hoyer, Brotman, Chan, & Needham, 2015). Increasing inpatients’ physical activity levels involves multiple actors, but nurses have a vital role in physical activity promotion due to the high amount of patient contact hours and the nature of their profession (Wald et al., 2019). Based on the education and professional profile, nurses are expected to signal risks and perform tasks to promote physical activity during hospitalization (Canadian Nurses association, 2015; Commissie kwalificatiestructuur, 1996; International Council of Nurses, 2018; Nursing & Midwifery Board of Australia, 2010; The Nursing & Midwifery Council, 2010; Tucker & Carr, 2016). However, it was indicated that nurses infrequently initiate physical activity during hospitalization (Doherty‐King, Yoon, Pecanac, Brown, & Mahoney, 2014; Lamarche & Vallance, 2013).

Therefore, it is necessary to understand their perception of their role in physical activity promotion and the factors influencing physical activity promotion.

## 2 BACKGROUND

Multiple interventions targeting physical activity promotion in older hospitalized patients have been developed, but the results of such interventions were inconsistent (Scheerman, Raaijmakers, Otten, Meskers, & Maier, 2018). Implementation of interventions in clinical practice is challenging since it is affected by factors on organizational, professional, intervention and patient level (Chaudoir, Dugan, & Barr, 2013). Previous studies have developed mapping guides addressing factors for implementing in hospital physical activity interventions including knowledge, attitudes and barriers as perceived by various healthcare providers and patients (Moore et al., 2014; Zisberg et al., 2018). Moore et al. (2014) concentrated on barriers to behaviour change using the “capability, opportunity, motivation‐behaviour (COM‐B) system.” Zisberg et al. (2018) used a human factors framework concentrating on the perception and role of involved actors in physical activity and environmental and organizational factors. However, nurses were included in both studies, the perception of nurses on barriers in physical activity promotion was not extensively addressed. Both quantitative (Dermody & Kovach, 2017; Elo, Saarnio, Routasalo, & Isola, 2012) and qualitative (Boltz et al., 2011; Chan, Hong, Tan, & Chua, 2019) approaches have been used to explore the nurses’ perspective on physical activity and strategies to increase physical activity levels of hospitalized patients. Using a questionnaire survey, Dermody and Kovach (2017) focused on knowledge, attitude and external barriers in physical activity promotion and Elo et al. (2012) concentrated on the whole rehabilitation process of older patients in an acute hospital setting and highlighted the opportunity for nurses to have an active role in rehabilitation teams. Focus groups were conducted by Boltz et al. (2011) to examine beliefs about activities of daily living and the perceived barriers and enablers, while Chan et al. (2019) focussed on patients' participation in physical activity. In our study, we used a mixed method sequential explanatory approach and specifically concentrate on the nurse perspective on their own role and the role of others, in physical activity promotion and factors influencing their behaviour. The central research question of our study is as follows: How do nurses perceive tasks and responsibilities in promoting physical activity and what factors are of influence on their physical activity promotion in older patients during hospitalization?

## 3 METHODS

### 3.1 Design

This mixed methods sequential explanatory (Pluye & Hong, 2014) study encompassed a quantitative and a qualitative component including questionnaire surveys (March–July 2016) addressing the nurses’ role in physical activity promotion and the factors that might be of influence and in‐depth interviews (June–August 2017) allowing us to further explore the nurses’ perception on physical activity promotion. The study was conducted at an academic teaching hospital in The Netherlands. The study is presented following the COREQ checklist, see Supplementary File 1. Table 1 gives an overview of the study design and methodology used in both components.

**Table 1:** Overview of study design and methodology

	Questionnaire component	Interview component
Aim and approach	Quantitative approach to explore the nurses’ perception on their role in physical activity promotion and on influencing factors.	Quantitative and qualitative approach to explore the nurses’ perception on specific tasks and responsibilities in physical activity promotion and on the most important factors influencing physical activity promotion Interview design was based on the results of the questionnaire component.
Study period	March‐July 2016	June‐August 2017
Target group	Nurse students, nurses, nurse supervisors	Nurses
Wards	All wards, except medium/intensive care	Internal medicine, traumatology, oncological surgery and a combined ward of vascular surgery, nephrology and urology
Participant selection	Numerical lot drawing from staff lists	Numerical lot drawing from staff lists
Topics and structure	Participant characteristics (quantitative) Perception on physical activity and on role in physical activity promotion (quantitative) Factors influencing physical activity promotion categorized in characteristics of the professional, patient, organization or intervention, and social factors (quantitative)	Participant characteristics (quantitative) Perception on physical activity and on specific tasks and responsibilities in physical activity promotion (quantitative and qualitative) Factors influencing physical activity promotion categorized in characteristics of the professional, patient, organization or intervention, and social factors (qualitative)
Analysis	Descriptive analysis	Descriptive analysis for quantitative data and deductive approach with directed content analysis for qualitative data

### 3.2 Participants

Nurse students, nurses and nurse supervisors were eligible when they were 18 years and older, were working on wards providing care to patients 70 years and older and had provided care to at least one patient of 70 years or older in the previous month. Nurses were considered nurse supervisors when they had a hierarchical position (team leader) on the ward. All wards, except medium/intensive care was included for the questionnaire component. Included wards for the interview component were as follows: internal medicine; traumatology; oncological surgery; and a combined ward of vascular surgery, nephrology and urology. For both questionnaires and interviews, nurses were selected from staff lists using a numerical lot drawing performed by a researcher. Participation in both the questionnaire as well as interview component was allowed. Nurse students, nurses and nurse supervisors were included as distinct groups in the questionnaire component to explore possible differences in their perception on physical activity promotion. For the interview component, only nurses were included. Sample size was determined based on those of similar studies (Boltz et al., 2011; Chan et al., 2019) since power analysis was not possible.

### 3.3 Data collection

#### 3.3.1 Questionnaire component

A questionnaire was developed to investigate the nurses’ role in physical activity promotion and factors influencing physical activity promotion during hospitalization of older patients as perceived by nurses. The head of the ward or a researcher contacted the selected nurses in person or by e‐mail, handed out the questionnaire surveys and monitored if the survey was completed. Questionnaire completion took approximately 20 min.

In the questionnaire, characteristics of the participants and two main topics were addressed: (a) perception on physical activity and their role in physical activity promotion and (b) factors influencing physical activity promotion. The following data were collected: age, gender, educational level, work experience, education completed on physical activity promotion (yes/no), self‐perceived motivation to promote physical activity (sufficient, yes/no), self‐perceived level of knowledge of physical activity promotion (sufficient, yes/no), satisfaction of level of physical activity promotion of the ward (sufficient, yes/no), satisfaction of level of physical activity promotion by physicians (sufficient, yes/no), satisfaction of level of physical activity of the patient (sufficient, yes/no) and perceived responsibilities in physical activity promotion. To explore the nurse's perception on their role, nurses were asked if they considered (a) daily activities (e.g. teeth brushing at the sink and walking towards the toilet), (b) unsupervised additional physical activity (e.g. stretch and gait exercises), (c) additional physical activity supervised by a nurse and (d) additional physical activity supervised by other healthcare professionals as physical activity during hospitalization and whether they promote daily activities, additional physical activity or consulted other healthcare professionals. Furthermore, nurses were asked to score the importance of 34 literature‐based factors in physical activity promotion of older patients during hospitalization using a Likert scale from 1 (totally not important) to 5 (very important) and to indicate their overall most important factor. The factors were selected based on previously indicated barriers in physical activity promotion in other studies with similar target groups or settings and were categorized in characteristics of the professional, patient, organization or intervention and social factors (Bonner & Sando, 2008; Chaudoir et al., 2013; Fleuren, Wiefferink, & Paulussen, 2004; Godin, Belanger‐Gravel, Eccles, & Grimshaw, 2008; Grol & Grimshaw, 2003; Huijg et al., 2015; Kajermo et al., 2008; Maue, Segal, Kimberlin, & Lipowski, 2004; Ploeg, Davies, Edwards, Gifford, & Miller, 2007). All factors are presented in Figure S1.

#### 3.3.2 Interview component

Targeted semi‐structured interviews, based on the outcomes of the questionnaires, were conducted to gain in‐depth information on nurses’ perspective on specific task and responsibilities of different actors in physical activity promotion and factors influencing physical activity promotion. The selected nurses were contacted in person or by e‐mail by the head of the ward or by the researcher. Interviews were held in private rooms and had a duration of 30–50 min. The interviews were held by one researcher, the first ten interviews were supervised by a second researcher. The interviewers had a background in health sciences or medicine and were trained and had experience in conducting interviews. They had no personal relationship with the participants and were introduced as independent researchers. The interviews were audio recorded and notes were made. The semi‐structured interview design contained questions of both quantitative and qualitative nature.

In the interviews, characteristics of the participants and two main topics were addressed: (a) perception on physical activity and specific tasks and responsibilities in physical activity promotion and (b) most important factors influencing physical activity promotion. A patient case was provided at the start of the interview to eliminate ward‐specific elements, describing a 82‐year‐old woman with urosepsis, who had high fever and showed signs of delirium at admission, progressively got better at days two of admission and almost completely recuperated at day five of admission. Nurses were asked to define physical activity during hospitalization, to score the importance of physical activity promotion on a Visual Analogue Scale and to describe how satisfied they were with the level of physical activity promotion during hospitalization. Tasks and responsibilities of nurses, physiotherapists, occupational therapists, physicians, patients and carers were specified with questions on the nurses’ perspective on responsibilities in signalling and performing different physical activity promotion tasks ((a) transfer from bed to chair, (b) activities of daily living, (c) supervised additional physical activity and d. unsupervised additional physical activity). Nurses were also asked to motivate which actor they thought to have final responsibility regarding these tasks and if these responsibilities would change when a patient is able to but is not performing physical activity during hospitalization. The nurse's perception on most important factors influencing physical activity promotion was explicitly discussed at the end of the interview but could be addressed during the whole interview. A printed copy of the 34 factors (see supplementary file 2) was handed out to the participants.

### 3.4 Data analysis

#### 3.4.1 Questionnaire component

Statistics were performed using IBM SPSS statistics version 22.0. Data were expressed as number and percentages. Factors were considered important when Likert scores exceeded four representing “important” and “very important.” Fishers exact test was used to analyse differences between nurse students, nurses and nurse supervisors.

#### 3.4.2 Interview component

Descriptive analyses of quantitative data (nurse characteristics and tasks and responsibilities) were performed using IBM SPSS statistics version 22.0. For qualitative analyses (perception on physical activity promotion, tasks and responsibilities and most important factors), a deductive approach (Elo & Kyngas, 2008) with directed content analysis was used (Hsieh & Shannon, 2005).

The interviews were fully transcribed. Initial codes and categories were based on the main topics of the interview design. Interview transcripts were read through, and initial coding was assigned to the correlating text. The categorization matric with pre‐determined categories and codes is presented in Table S1. Subsequently, open coding was used to determine possible new codes and categories for data not corresponding with the pre‐determined codes. Atlas.ti 8.0 was used in the qualitative coding process. Discussions on interpreting data took place between two researchers, and all codes and data were verified by a second researcher.

### 3.5 Validity and reliability

The self‐developed questionnaire and interview design were designed based on literature review and expert opinion. To increase validity, a group of experts, consisting of physicians specialized in geriatrics, general medicine and rehabilitation medicine, a nurse director, a nurse specialized in geriatrics and an advisor in quality and safety of health care, compiled and critically revised both the survey and interview design. In addition, structure and content validity were discussed with a group of PhD students and professors in medicine and behavioural sciences. Subsequently, the questionnaire survey and interview design were tested and refined with four nurses, working at the hospital on different wards and with different levels of work experience, by pilot testing on feasibility and nurses’ interpretation. As a result, the description of factors in the questionnaire and the description of the patient case and sequence of questions in the interview design were adjusted. To determine reliability, the questionnaire and interview design were re‐evaluated in a second pilot with two other nurses, also working at the hospital. To limit bias, nurses were enabled to fill in the questionnaire survey anonymously at any desired moment and the interviews were conducted in a private setting whereas the independent position of the interviewer was emphasized.

### 3.6 Ethics

The study was approved by the medical ethical committee of a Dutch academic teaching hospital (2016.255). All enrolled nurses provided written informed consent.

## 4 RESULTS

Thirteen nurse students, 85 nurses and ten nurse supervisors participated in the questionnaire component of the study. In the interview component, three nurses refused to participate resulting in a total of 51 interviews. The characteristics of all participants were shown in Table 2. In both study components, 86% of the nurses was female, and the nurses had a comparable median age (questionnaire: 32 years; interview: 31 years) and level of work experience (questionnaire: 6 years; interview: 7 years). In the questionnaire component, more nurses had a high educational level compared with the interview component (57.6% vs. 47.1%). A minority of nurse students (38.5%), nurses (20.0%) and nurse supervisors (30.0%) received education on physical activity promotion in the prior year.

**Table 2:** Characteristics of the study population for both questionnaires and interviews, stratified by nurse student, nurse and nurse supervisor

	Questionnaire (*N* = 108)		Interview (*N* = 51)	
	Nurse student *N* = 13	Nurse *N* = 85	Nurse supervisor *N* = 10	Nurse *N* = 51
Age, years, median [IQR]	25 [23.0–29.0]	32 [25.0–51.0]	49 [42.8–54.5]	31 [26.0–45.0]
Female, *N* (%)	11 (84.6)	73 (85.9)	10 (100)	44 (86.3)
Educational level high^a^, *N* (%)	7 (53.8)	49 (57.6)	10 (100)	24 (47.1)
Work experience nurse, y, median [IQR]	N/A	6 [3.0–20.0]	25 [19.3–30.3]	7 [2.5–18.0]
Work experience as nurse supervisor, y, median [IQR]	N/A	N/A	7 [5.5–10.0]	N/A
Education PA promotion^b^, yes, *N* (%)	5 (38.5)	17 (20.0)	3 (30.0)	N/a

Abbreviations: IQR, interquartile range; N/A, not applicable; N/a, not available; PA, physical activity.

^a^ High educational level defined as applied university and higher.

^b^ Additional education on PA promotion in the previous year.

### 4.1 Tasks and responsibilities in physical activity promotion

The questionnaire component revealed that nurses feel responsible (89.4%) to promote physical activity during hospitalization. As presented in Table 3, nurses promote daily activities (95.3%) and consult other healthcare professionals to promote physical activity (90.6%). Seventy‐eight per cent of the nurses stated to actively promote additional physical activity like stretch and gait exercises. Only one nurse reported to not promote physical activity at all. No differences were observed between nurse students, nurses and nurse supervisors. Eighty‐seven per cent of the respondents stated that physiotherapists, physicians or the patients had responsibilities in physical activity promotion. Occupational therapists (13%) and carers (22%) were also mentioned to be responsible.

**Table 3:** Perception of physical activity promotion during hospitalization and methods to promote physical activity, stratified by nurse student, nurse and nurse supervisor (questionnaire component, *N* = 108)

	Nurse student *N* = 13	Nurse *N* = 85	Nurse supervisor *N* = 10
Perception of physical activity promotion, *N* (%)			
Motivated to promote PA, yes	12 (92.3)	83 (97.6)	10 (100)
Responsible to promote PA, yes	13 (100)	76 (89.4)	10 (100)
Self‐perceived level of knowledge about PA promotion, sufficient	9 (69.2)^a^	77 (90.6)	8 (80.0)
PA promotion on ward, present	8 (61.5)	45 (52.9)	6 (60.0)
Satisfied with PA promotion on ward, yes	9 (69.2)	56 (65.9)	7 (70.0)
Satisfied with PA promotion by physicians, yes	5 (38.5)	41 (48.2)	6 (60.0)
Satisfied with level of PA of patient, yes	4 (30.8)	27 (31.8)	4 (40.0)
Definition of physical activity during hospitalization, *N* (%)			
Daily activities	12 (92.3)	68 (80.0)	8 (80.0)
Unsupervised additional PA of patient	10 (76.6)	66 (77.6)	8 (80.0)
Supervised additional PA with nurse	11 (84.6)	70 (82.4)	8 (80.0)
Supervised additional PA with other healthcare professionals	12 (93.2)	68 (80.0)	8 (80.0)
Methods of physical activity promotion during hospitalization, *N* (%)			
Promote daily activities	12 (92.3)	81 (95.3)	10 (100)
Promote additional PA (e.g. stretch and gait exercises)	9 (69.2)	66 (77.6)	8 (80.0)
Consult other healthcare professionals to promote PA	11 (84.6)	77 (90.6)	10 (100)
I do not promote PA	0 (0)	1 (1.2)	0 (0)

Abbreviation: PA, physical activity.

^a^ Difference between nurse student, nurse and nurse supervisor (*p* < .05).

**FIGURE 1:**
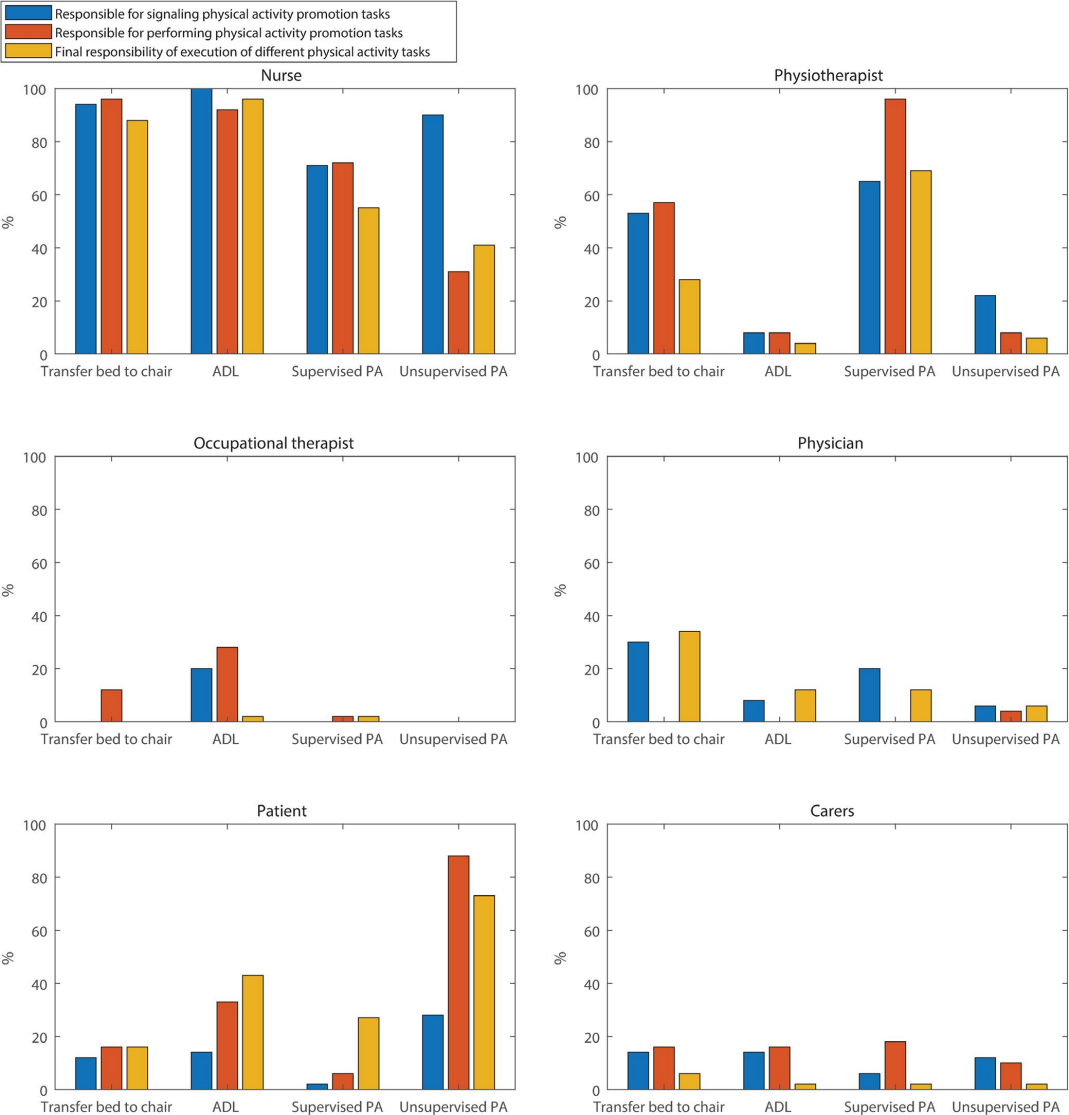
Tasks and responsibilities of different actors in physical activity promotion during hospitalization according to nurses (%) (interview component, *N* = 51)

During the interviews, nurses stated to be responsible for signalling and performing physical activity promotion tasks and had final responsibility for transfers from bed to chair and promotion of daily activities. Nurses indicated that physiotherapists have a greater responsibility of supervised additional physical activity and it is the patients’ responsibility to do unsupervised additional physical activity. The majority of the nurses stated that patients’ responsibilities increase when patients become more independent during hospital admission and that they would motivate patients to be physically active by providing information on consequences of physical inactivity and discussing the reasons for the patients’ physical inactivity. *“You try to explore why someone does not want to be physically active and if you know the reason, you can advise. If it is fear, you try to overcome it or possibly use an aid to provide more certainty… you can explain it again and again, but eventually it is their responsibility.”* The tasks and responsibilities of the physician were described as to determine the patient's ability to perform different levels of physical activity and to motivate patients when they refuse to perform physical activity. *“The physician should assess when activities like walking the stairs can be performed, I think that's up to them.”* The different tasks and responsibilities in physical activity promotion of all actors according to nurses are visualized in Figure 1.

### 4.2 Factors influencing physical activity promotion

Sixty‐six per cent of nurses of the questionnaire component were satisfied with physical activity promotion on their ward, 48% were satisfied with physical activity promotion by physicians and 32% were satisfied with the actual level of physical activity of the patients during hospitalization. An overview of the importance of factors influencing physical activity promotion during hospitalization is provided in Table 4. Differences in importance between nurse students, nurses and nurse supervisors were observed for the factors “Fear of falling during PA promotion,” “Availability of protocol” and “Opinion towards PA promotion of colleagues.”

**Table 4:** Importance of factors (Likert score > 3) influencing physical activity promotion in older patients during hospitalization, stratified by nurse student, nurse and nurse supervisor (questionnaire component, *N* = 108)

	Importance (Likert score > 3)				Fisher's exact test	Rated as most important (%)
	Overall (*N* = 108)	Nurse student (*N* = 13)	Nurse (*N* = 85)	Nurse supervisor (*N* = 10)		
Characteristics of the professional, *N* (%)						
Motivation for PA promotion	96 (88.9)	11 (84.6)	76 (89.4)	9 (90.0)	0.854	2.8
Knowledge of methods of PA promotion	97 (89.8)	12 (92.3)	75 (88.2)	10 (100)	0.845	0.9
Knowledge of importance of PA promotion	99 (91.7)	11 (84.6)	78 (91.8)	10 (100)	0.448	7.4
Fear of loss of function when PA is not promoted	66 (61.1)	9 (69.2)	51 (60.0)	6 (60.0)	0.882	1.9
Fear of decubitus ulcer when PA is not promoted	85 (78.7)	10 (76.9)	68 (80.0)	7 (70.0)	0.704	0.9
Fear of falling during PA promotion	43 (39.8)	2 (15.4)	39 (45.9)	6 (60.0)	0.049^a^	–
Characteristics of the patient, *N* (%)						
Admission diagnosis	81 (75.0)	7 (53.8)	68 (80.0)	6 (60.0)	0.068	4.6
Comorbidity	81 (75.0)	8 (61.5)	65 (76.5)	8 (80.0)	0.483	2.8
Delirium	86 (79.6)	11 (84.6)	66 (77.6)	9 (90.0)	0.758	–
Dementia	48 (44.4)	4 (30.8)	38 (44.7)	6 (60.0)	0.393	–
Pain	96 (88.9)	13 (100)	74 (87.1)	9 (90.0)	0.466	7.4
Physical constraints	98 (90.7)	11 (84.6)	77 (90.6)	10 (100)	0.494	2.8
Motivation performing PA	90 (83.3)	12 (92.3)	72 (84.7)	6 (60.0)	0.106	11.1
Self‐efficacy performing PA	83 (76.9)	10 (76.9)	66 (77.6)	7 (70.0)	0.846	0.9
Ethnic background	40 (37.0)	2 (15.4)	34 (40.0)	4 (40.0)	0.249	–
Language barrier	48 (44.4)	4 (30.8)	41 (48.2)	3 (30.0)	0.346	–
Characteristics of the organization, *N* (%)						
Availability of equipment	107 (99.1)	13 (100)	84 (98.8)	10 (100)	1.000	2.8
User friendliness of equipment	104 (96.3)	12 (92.3)	82 (96.5)	10 (100)	0.622	–
Staffing ratio	102 (94.4)	12 (92.3)	80 (94.1)	10 (100)	0.771	14.8
Workload	83 (76.9)	8 (61.5)	68 (80.0)	7 (70.0)	0.269	9.3
Physical environment of ward	86 (79.6)	8 (61.5)	69 (81.2)	9 (90.0)	0.197	1.9
Educational support for PA promotion	73 (67.6)	7 (53.8)	57 (67.1)	9 (90.0)	0.197	0.9
Characteristics of the intervention, *N* (%)						
Availability of protocol	41 (38.0)	1 (7.7)	35 (41.2)	5 (50.0)	0.038^a^	–
Clarity of instructions of protocol	50 (46.3)	4 (30.8)	41 (48.2)	5 (50.0)	0.515	–
Evidence based practice	53 (49.1)	5 (38.5)	42 (49.4)	6 (60.0)	0.615	–
Availability of information materials	77 (71.3)	6 (46.2)	62 (72.9)	9 (90.0)	0.062	–
PA promotion incorporated in daily work routine	92 (85.2)	11 (84.6)	71 (83.5)	10 (100)	0.540	–
Time investment in PA promotion	90 (83.3)	11 (84.6)	69 (81.2)	10 (100)	0.459	0.9
Visible progress after PA promotion	88 (81.5)	9 (69.2)	70 (82.4)	9 (90.0)	0.447	–
Social factors, *N* (%)						
Culture PA promotion at ward	94 (87.0)	12 (92.3)	73 (85.9)	9 (90.0)	1.000	–
PA promotion by physician	88 (81.5)	9 (69.2)	70 (82.4)	9 (90.0)	0.447	–
Opinion towards PA promotion of colleagues	63 (58.3)	4 (30.8)	50 (58.8)	9 (90.0)	0.016^a^	–
Influence of cares on PA promotion	100 (92.6)	12 (92.3)	79 (92.9)	9 (90.0)	0.818	–
Professional patient‐nurse relationship	56 (51.9)	6 (46.2)	46 (54.1)	4 (40.0)	0.694	–

Abbreviations: PA, physical activity.

^a^ Difference between nurse student, nurse and nurse supervisor (*p* < .05).

#### 4.2.1 Characteristics of the professional

Almost all nurses scored knowledge of methods of physical activity promotion (88.2%), knowledge of importance of physical activity promotion (91.8%) and nurse’ motivation (89.4%) as important factors influencing physical activity promotion (Table 4). Nurses perceived their level of knowledge of physical activity promotion as sufficient (90.6%) in contrast to 69.2% of the nurse students (Table 3). Nurses were motivated to promote physical activity (97.6%) and considered daily activities (80.0%) and additional physical activity supervised by a nurse (82.4%) or other healthcare professional (80.0%) as physical activity during hospitalization (Table 3). Unsupervised additional physical activity was not seen as physical activity during hospitalization by 22.4% of the nurses. No differences were observed between the nurse students, nurses and nurse supervisors.

During the interviews, nurses described physical activity during hospitalization as: *“out of bed,” “walking,” “movement in bed” and “sitting in chair.”* Nurses scored the importance of physical activity during hospitalization with a median VAS score of 8.9 [5.5–10.0]. Most frequently named motivations were preventing risk of complications (e.g. decubitus and pneumonia), loss of muscle mass and slower recovery. Long‐term adverse outcomes like functional loss and regaining self‐reliance were named less frequent compared with more immediate noticeable adverse outcomes. Nurses stated they were not always motivated to promote physical activity, because they were empathic towards patients facilitating comfort rather than physical activity. *“You often feel sorry for older people and well, if they want to stay in bed a day longer… for example, a patient of 92 years and oh well, you allow her to stay in bed for the day*”. Also, the need for physical activity promotion in older patients was questioned. Nurses stated to understand the low motivation of older patients when being sick, had a bad night sleep or being tired after a day with multiple examinations.

#### 4.2.2 Characteristics of the patient

Physical constraints (90.6%), pain (87.1%) and motivation of the patient (84.7%) were identified as important factors in physical activity promotion (Table 4). The factor “motivation performing physical activity” was scored as most important by 11.1% of the nurses.

During the interviews, patient motivation was stated as a barrier by 65% of nurses. Tiredness, pain, lines like catheters and drip lines, their previous inactivity at home and the belief it is uncommon to be physically active at an older age were explanations for a low motivation of patients. In addition, the patient’ perception of the hospital admission (47%) as a place to rest, be sick and were it is justified to be physically inactive was identified as factor influencing the patient’ level of physical activity during hospitalization: *“I have noticed that patients, when admitted to the hospital, even the patients who are mobile, immediately have the idea that they have to stay in bed all day in their pajamas.”* Seventy‐three per cent of the nurses suggested hospitals to focus on increasing patient awareness on importance of physical activity, for example using a flyer or video.

#### 4.2.3 Characteristics of the organization

Sufficient staffing ratio (94.1%) and availability of equipment (98.8%) were identified as important factors in physical activity promotion (Table 4). The factor “staffing ratio” was scored as most important by 14.8% of the nurses.

Nurses in the interview group stated that in case of a high number of complex patients (e.g. patients with delirium, physical impairments or multiple drip lines), workload and staffing ratio become barriers. Priorities shift when nurses experienced a low staff ratio and a high workload; physical activity was regarded as one of the first activities to be dropped and nurses stated that concessions were made in their physical activity promotion (e.g. use of bed urinal). *“...sometimes it is due to a high workload. You know it is important and benefits the patient, but you don't have enough time unfortunately… when other things have to be done, it is not a priority.”* Nurses suggested hospitals to invest in more staff (physiotherapists and medical students), equipment and adjustment of patient rooms to make them more attractive for physical activity. A living room, walking routes and activity counselling were other suggestions to increase physical activity of the older patients during hospitalization.

#### 4.2.4 Characteristics of the intervention and social factors

Social factors like influence of carers (92.9%), culture of physical activity promotion on ward (85.9%) and physical activity promotion by the physician (82.4%) were indicated as important factors in physical activity promotion. Availability (41.2%) and clarity (48.2%) of a protocol regarding physical activity promotion were scored less frequently as important factor in the questionnaire component (Table 4).

## 5 DISCUSSION

Nurses perceive to have a dominant role in physical activity promotion and feel responsible; however, they were not satisfied with the actual level of physical activity of older patients during hospitalization. Low patient motivation and priority shifts of tasks due to high workload were indicated as barriers. In addition, the role of physicians was indicated to be important to influence physical activity promotion behaviour.

### 5.1 Tasks and responsibilities in physical activity promotion

This study indicated that nurses must adopt various roles in physical activity promotion during older patients’ hospital admissions. Besides signalling and supporting physical activity promotion tasks and consulting other healthcare professional, nurses have an important role in motivating patients. Motivating patients and supporting self‐management become more prominent in nursing (The Nurse & Midwifery Council, 2018; V&V, 2020, 2012). However, nurse activities in physical activity promotion during hospitalization seem to target prevention of potential harm more than supporting rehabilitation goals (Kneafsey, Clifford, & Greenfield, 2013). Besides their own responsibilities in physical activity promotion, nurses named the responsibilities of patients themselves and of physical therapists in supervised activity like stretch and gait exercises. Zisberg et al. (2018) also indicated the multidisciplinary effort in mobilizing patients; however, greater responsibilities, higher knowledge and a more positive attitude towards mobility were assigned to physical therapists than to nurses. Nurses who believed to be responsible for ambulating patients during hospitalization rather than others were found to proactively address barriers concentrating on improving patient functional independence (Doherty‐King & Bowers, 2013). The reserved attitude of a part of the nurses in our study regarding physical activity of older patients during hospitalization and unsupervised physical activity suggests that the perception on physical activity during hospitalization needs further attention.

### 5.2 Factors influencing physical activity promotion

The identified factors influencing physical activity promotion by nurses, most importantly patient motivation and high workload causing priority shifts of tasks, are in line with previous studies addressing barriers in physical activity promotion during hospitalization (Boltz et al., 2011; Brown et al., 2007; Dermody & Kovach, 2018; Moore et al., 2014). The nurses in our study suggested to increase patient awareness on the importance of physical activity. Barriers for being physically active during the hospital admission from a patient perspective were previously addressed (Brown et al., 2007; Koenders et al., 2020), but better understanding of what causes low patient motivation is important.

According to the nurses in our study, physical activity promotion tasks became less of a priority when nurses experienced a low staff ratio and a high workload. Staff ratio and workload are associated with nursing tasks being left undone which was found to be related to the nurses’ perception of quality of nursing care (Ball, Murrells, Rafferty, Morrow, & Griffiths, 2014). In addition, an increase in nurses’ workload was found to affect patient outcomes (Aiken et al., 2014). This implies that nurse staffing levels should be increased or tasks must shift towards other actors or targeted by eHealth interventions (Jonkman, van Schooten, Maier, & Pijnappels, 2018). However, intervention studies on physical activity promotion of hospitalized patients showed positive results on physical activity levels using preexisting staff ratios (Hoyer et al., 2016; Liu et al., 2018).

In the current study, physical activity promotion by the physician and carers involvement was indicated as influencing factors. Physicians were expected to indicate the ability of patients to be physical active. Physicians orders regarding physical activity are found to influence patients’ decisions to perform physical activity during hospitalization (So & Pierluissi, 2012) but are infrequently discussed (So & Pierluissi, 2012) and often bed rest orders during hospital admission do not have valid and specified reasons (Brown et al., 2004). Awareness of the role of physicians in physical activity promotion might contribute to strengthen nurses’ physical activity promotion behaviour and increase physical activity levels of older hospitalized patients. Furthermore, carers could play a more prominent role in physical activity promotion. In our study, the role of carers in physical activity promotion during hospitalization was indicated as minor, but including carers in physical activity promotion of older patients was previously emphasized (Boltz et al., 2011).

### 5.3 Limitations

Evidence for the need of interventions targeting physical activity during hospitalization is growing. In this study, the perspective of nurses on tasks and responsibilities and factors influencing physical activity promotion during hospitalization was extensively addressed providing essential information for the development and implementation of in hospital physical activity interventions.

We included a large group of nurses and used a mixed methods approach to deepen the understanding of barriers in physical activity promotion by nurses. The questionnaire and interview design were self‐developed and not cross validated. The use of another validated instrument was not possible while there was none available for this specific interest. However, both questionnaire and interview design were based on literature and tested in advance on feasibility and interpretation of the questions. Due to the small number of participants included in the groups nurse students and nurse supervisors in the questionnaire component, we cannot conclude on the difference in perception on physical activity promotion between the three groups, although no significant differences were found.

## 6 CONCLUSION

Nurses perceive to have various roles in physical activity promotion and feel responsible, but they were not satisfied with the level of physical activity of patients. Contributing factors were low patient motivation and priority shifts due to high workload. Hospital managers and healthcare professionals should be aware of the various roles of nurses in physical activity promotion. Emphasis should be on the multidisciplinary approach of physical activity promotion including physicians, patients and carers.

## CONFLICT OF INTEREST

None of the authors reported any conflict of interest.

## AUTHOR CONTRIBUTIONS

KS, JM, JB, CM and AM: Substantial contributions to conception and design, or acquisition of data, or analysis and interpretation of data; drafting the manuscript or revising it critically for important intellectual content; final approval of the version to be published; agreeing to be accountable for all aspects of the work in ensuring that questions related to the accuracy or integrity of any part of the work are appropriately investigated and resolved. Each author should have participated sufficiently in the work to take public responsibility for appropriate portions of the content.

## Supporting information

Fig S1

Table S1

File 1
