# Supplementary figures and images for: Tasks and responsibilities in physical activity promotion of older patients during hospitalization: A nurse perspective
Source: Nurs Open. 2020 Aug 30;7(6):1966–77. doi: 10.1002/nop2.588 (PMC7544836; doi:10.1002/nop2.588)

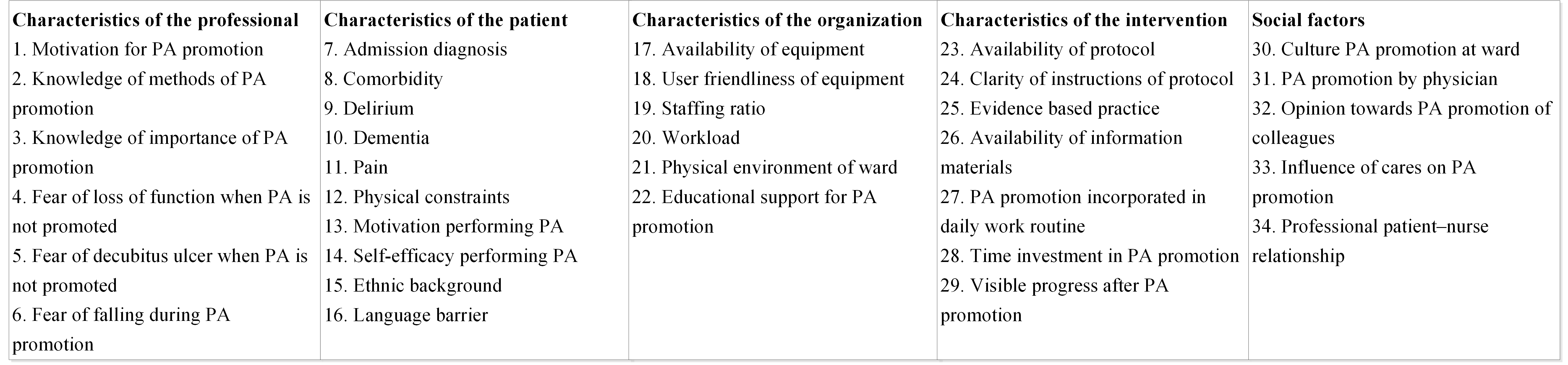

Supplement: Supplementary file 1 — Fig S1 [file NOP2-7-1966-s001.tif]
