# Supplementary material for: Tasks and responsibilities in physical activity promotion of older patients during hospitalization: A nurse perspective
Source: Nurs Open. 2020 Aug 30;7(6):1966–77. doi: 10.1002/nop2.588 (PMC7544836; doi:10.1002/nop2.588)
Supplement: Supplementary file 2 — Table S1 [file NOP2-7-1966-s002.docx]

Supplementary Table 1: Categorization matrix

| **Category** | **Subcategory** | **Pre-determined codes** |
| --- | --- | --- |
| Tasks and responsibilities | - | Definition of PA |
|  | - | Importance of PA |
|  | - | Level of PA |
|  | - | Perception on PA |
|  | Signaling | Transfer bed to chair, ADL, with help, without help |
|  | Performing | Transfer bed to chair, ADL, with help, without help |
|  | Final responsibility | Transfer bed to chair, ADL, with help, without help |
|  | Actors | Nurse, physical therapist, physician, dietician, occupational therapist, patient, carer |
| Factors | Characteristics of the professional | Motivation for PA promotion, knowledge of methods of PA promotion, knowledge of importance of PA promotion, fear of loss of function when PA is not promoted, fear of decubitus ulcer when PA is not promoted,fFear of falling during PA  promotion |
|  | Characteristics of the patient | Admission diagnosis, comorbidity, delirium, dementia, pain, physical constraints, motivation performing PA, self-efficacy performing PA, ethnic background, language barrier |
|  | Characteristics of the organization | Availability of equipment, user friendliness of equipment, staffing ratio, workload, physical environment of ward, educational support for PA promotion |
|  | Characteristics of the intervention | Availability of protocol, clarity of instructions of protocol, evidence based practice, availability of information materials, PA promotion incorporated in daily work routine, time investment in PA promotion, visible progress after PA promotion |
|  | Social factors | Culture PA promotion at ward, PA promotion by physician, opinion towards PA promotion of colleagues, Influence of cares on PA promotion, professional patient–nurse relationship |

PA: physical activity
